# Dose-Dependent Prophylactic Efficacy of Filarial Antigens Glutathione-S-Transferase and Abundant Larval Transcript-2 against *Brugia malayi* Challenge in *Mastomys*

**DOI:** 10.1155/2024/4543922

**Published:** 2024-07-26

**Authors:** Mohini Rambhau Nakhale, Priyanka Bhoj, Namdev Togre, Vishal Khatri, Lalit Batra, Udaikumar Padigel, Kalyan Goswami

**Affiliations:** ^1^ Department of Biochemistry JB Tropical Disease Research Centre Mahatma Gandhi Institute of Medical Sciences, Sevagram 442 102, Maharashtra, India; ^2^ Department of Pathology and Laboratory Medicine Lewis Katz School of Medicine Temple University, Philadelphia 19140, PA, USA; ^3^ Regional Biocontainent Laboratory Center for Predictive Medicine for Biodefense and Emerging Infectious Diseases University of Louisville, Louisville 40222, KY, USA

## Abstract

**Objective:**

To identify the most effective dose of filarial r*Bm*ALT-2 and r*Wb*GST alone or in combination against *B. malayi* infection *in vitro* and *in vivo*.

**Methods:**

*Mastomys* (*n* = 5–7/group) received intramuscular (i.m.) injection with three different doses (25, 50, and 100 *μ*g) of r*Bm*ALT-2 or r*Wb*GST, either alone or in combination with alum as the adjuvant. Protective immunity was studied by *in vivo* and *in vitro* cytotoxicity assay. To evaluate the cellular immune response, splenocyte proliferation and cytokine profile were assessed.

**Results:**

Serological results revealed a substantial (*p* < 0.005) induction of IgG1, IgG2a, and IgG3 responses in vaccinated *Mastomys*. *Mastomys* immunized with 50 *μ*g r*Bm*ALT-2 + alum induced 79–81% killing against the L3 larvae challenge *in vivo* and *in vitro* ADCC assay (*p* < 0.005); whereas r*Wb*GST + alum alone or in combination with r*Bm*ALT-2 + alum induced 63–68% killing (*p* < 0.005) *in vivo* and *in vitro*. Antigen-specific cytokine profiles of *Mastomys* vaccinated with either *Bm*ALT-2, *Wb*GST or a combination showed elevated IL-10, IL-4, and IFN-*γ* levels, signifying both Th1 and Th2 immune response.

**Conclusions:**

These findings suggest that immunization of *Mastomys* with a 50 *μ*g/dose of r*Bm*ALT-2 + alum four times at a 4-week interval demonstrated considerable protection against *B. malayi* infection.

## 1. Introduction

Parasitic worms, specifically *Wuchereria bancrofti*, *Brugia malayi*, and *B. timori*, cause lymphatic filariasis (LF), influencing roughly 863 million individuals worldwide [1]. Mass drug administration (MDA) is one of the strategies used by the World Health Organization (WHO) in the Global Programme to Eliminate Lymphatic Filariasis, which aims to eradicate the disease by 2030 [1]. Since consistent MDA renders parasites vulnerable to developing drug resistance, several treatment modes should be considered. Prophylactic vaccination is one such approach that may aid in LF control and elimination worldwide [2]. Moreover, LF re-emergence in several countries has highlighted the importance of developing a prophylactic vaccine for LF [3–5].

Two filarial antigens, *B. malayi* abundant larval transcript-2 (*Bm*ALT-2) and *W. bancrofti* glutathione-S-transferase (*Wb*GST), have been proposed as promising vaccine candidates [6, 7]. The ALT-2 protein stimulates high protective immune responses against *B. malayi* [7–11]. The filariae express these proteins at the late second stage larvae2 (L2) and peak in the third stage larvae3 (L3) [12], playing a pivotal role in transmission and infectivity [8, 13]. The ALT-2 protein has no mammalian homologue [7, 10], and it is unique among host components and has been shown to have an immunomodulatory function in the primary host immune response [14, 15]. Studies suggest that ALT proteins from *B. malayi* are involved in activating Th2 immune responses that can aid in the survival of parasites [13]. GSTs are one of the crucial enzymes that efficiently inhibit the host's oxidative free radicals [7, 16]. GSTs, primary phase II detoxification enzymes, and antioxidants are crucial for parasite survival in the host [7–9, 13, 17]. Previous vaccination studies in Jirds and *Mastomys* using r*Bm*ALT-2 or r*Wb*GST antigens reported substantial protection against filariasis [7, 10, 18].

This study aimed to evaluate the effective dose of both antigens (r*Bm*ALT-2 and r*Wb*GST) either as a single or combination vaccine in *Mastomys coucha*.

## 2. Materials and Methods

### 2.1. Animals and Filarial Parasites

All experiments involving male *M. coucha* (males, 6–8 weeks) were carried out according to guidelines described by the National Institutes of Health (NIH) and Office of Laboratory Animal Welfare (OLAW) USA [19, 20] and Institutional Animal Ethics Committee (IAEC) of Mahatma Gandhi Institute of Medical Sciences (MGIMS), Sevagram, Maharashtra, India (MGIMS/IAEC/July/8/2014). Baermann's method was employed to obtain *B. malayi* infective L3 as described [21, 22] and used for further experimental procedures.

### 2.2. Protein Expression and Purification

The r*Bm*ALT-2 and r*Wb*GST proteins were expressed as previously described [8]. Briefly, the *bmalt-2* and *wbgst* genes in the pRSET-A vector were expressed in the BL21 (DE3) pLysS expression host. Isopropyl *β*-D-1-thiogalactopyranoside of 1 mM (IPTG; Merck Millipore, Bangalore, India) was used to induce these six-histidine-tagged recombinant proteins and further purified by nickel-charged sepharose column (Thermo Fisher Scientific, Mumbai, India). Limulus amebocyte lysate (LAL) chromogenic quantification kit (Thermo Fisher Scientific, Mumbai, India) was used to detect and eliminate endotoxins from purified protein preparations. Protein expression and purity were determined by 12% SDS-PAGE and western blotting with anti-6XHis antibodies (Qiagen, New Delhi, India).

### 2.3. Immunization, Challenge, and Sera Collection of *Mastomys*

**Figure 1:**
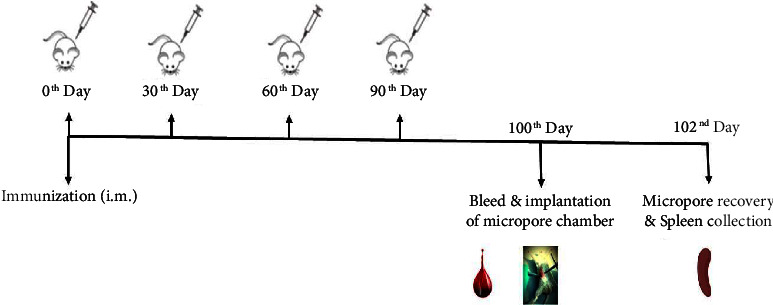
Representation of the intramuscular (i.m.) immunization protocol. *Mastomys* immunized i.m. with different doses (25, 50, and 100 *μ*g) of r*Bm*ALT-2, r*Wb*GST, or r*Bm*ALT-2 + r*Wb*GST with alum as adjuvant or alum only (control) at four weeks of interval.

*Mastomys* were divided into ten groups (5–7/group). Vaccinated groups received intramuscular (i.m.) injections of 25, 50, or 100 *μ*g/dose of the respective antigens plus alum as adjuvant four times at an interval of 4 weeks as follows: Groups 1, 2, and 3: *Mastomys* immunized with alum plus r*Bm*ALT-2-25 *μ*g, r*Bm*ALT-2-50 *μ*g, and r*Bm*ALT-2-100 *μ*g, respectively; Groups 4, 5, and 6: *Mastomys* immunized with alum plus r*Wb*GST-25 *μ*g, r*Wb*GST-50 *μ*g, and r*Wb*GST-100 *μ*g, respectively; Groups 7, 8, and 9: *Mastomys* immunized with alum plus r*Bm*ALT-2 + r*Wb*GST-25, 50, and 100 *μ*g (1 : 1 concentration), respectively; and Group 10: *Mastomys* administered with alum adjuvant alone (control group). Ten days after the last immunization sera were collected by intraorbital bleeding to be used for serum analysis for antibody titers. Vaccine-induced protection, cytotoxicity, were assessed by inserting a plexiglass diffusion chamber containing 20 *B. malayi* L3 (Millipore Sigma, Bedford, MA) into *Mastomys* peritoneal cavity as previously mentioned [10]. After 48 hr, the chambers were retrieved, and their contents were microscopically examined to assess larval killing and cell adhesion. The results were expressed as a percentage cytotoxicity value (Figure 1).

### 2.4. Splenocyte Proliferation and Cytokine Expression in Culture Supernatant

*Mastomys* were euthanized by an overdose of ketamine, and spleens were aseptically removed at day 102 (Figure 1), minced, and washed thrice using RPMI-1640 medium, along with 10% heat-inactivated FBS fetal calf serum (FBS) and 1×antibiotic/antimycotic solution (Sigma-Aldrich, Mumbai, India). Single-cell suspensions were prepared (2 × 10^6^ cells/well in 200 *μ*L RPMI media); stimulated with 1 *μ*g/well of the respective antigen (r*Bm*ALT-2 or r*Wb*GST), 1 *μ*g/well of positive control concanavalin A (ConA; Sigma-Aldrich, Mumbai, India) and negative control (unstimulated cells) were plated in triplicate in cell culture plates (96-well plate) and incubated for 48 hr (37°C in 5% CO_2_). The MTS assay kit (Promega, New Delhi, India) was used to measure cell proliferation, and the stimulation index (SI) was calculated as previously described [11].

For cytokine analysis, similar sets of single cell suspension (2 × 10^6^ cells/mL/well) were prepared and plated in cell culture plates (24-well plate), which were further stimulated with 10 *μ*g/well of the respective antigens or 2 *μ*g/well of ConA (positive control) and incubated for 72 h (37°C in 5% CO_2_). The cytokine ELISA kits (Invitrogen Bioservices, Bengaluru, India) were used to assess the amounts of IL-4, IL-10, and IFN-*γ* in the culture supernatants after incubation following the manufacturer's instructions.

### 2.5. Antigen-Specific IgG Titer and Isotype in the Sera of Vaccinated *Mastomys*

An indirect ELISA was used to quantify the amounts of r*Bm*ALT-2- or r*Wb*GST-specific total IgG antibodies in the sera of *Mastomys* [11]. The serum samples collected on day 100 (Figure 1) were diluted at dilutions of 1 : 100, 1 : 500, 1 : 1000, and 1 : 10000 and tested for the presence of IgG antibodies using a goat anti-mouse HRP-conjugated secondary antibody diluted 1 : 10000. (Thermo Fisher Scientific, Mumbai, India). TMB substrate was used to develop the reaction, and adding 2M H_2_SO_4_ stopped the color development. A spectrophotometer (Biotek, New Delhi) was used to measure the absorbance at 450 nm.

The concentrations of antigen-specific isotypes (such as IgG1, IgG2a, IgG2b, and IgG3) against r*Bm*ALT-2 or r*Wb*GST were measured in the blood samples using an indirect ELISA method, as explained in a previous study [11], with the use of the specific HRP-labeled antibodies for each isotype.

### 2.6. *In Vitro* Cytotoxicity Experiment Using *B. malayi* L3

To assess the cytotoxicity of antigen-specific protective antibodies against *B. malayi* L3, an *in vitro* cytotoxicity assay (antibody-dependent cellular assay; ADCC) was used as previously described [7]. Briefly, peritoneal exudate cells (PEC; 2 × 10^5^ cells/well) collected from nonimmunized *Mastomys* were incubated with 10–12 *B. malayi* L3 and 50 *μ*L pooled sera samples from immunized *Mastomys* into 96-well cell culture plates (Thermo Fisher Scientific, Mumbai, India) for 48 hr at 37°C in 5% CO_2_. After 48 hr incubation, the viability of L3 was checked, and percentage cytotoxicity was calculated as previously described [7].

### 2.7. Statistical Assessment

The statistical studies were conducted using SPSS V21.0 software (IBM, SPSS Inc., India). Survival data were examined using one-way analysis of variance (ANOVA) with a Bonferroni post hoc test. *p* values <0.05 were considered significant. Sera and cytokine were analyzed by the Kruskal–Wallis test followed by Bonferroni post-test multiple comparisons, *p* values <0.05 and < 0.005 were considered significant.

## 3. Results

### 3.1. All Immunized *Mastomys* Had a High Antigen-Specific IgG Antibody Titer

**Figure 2:**
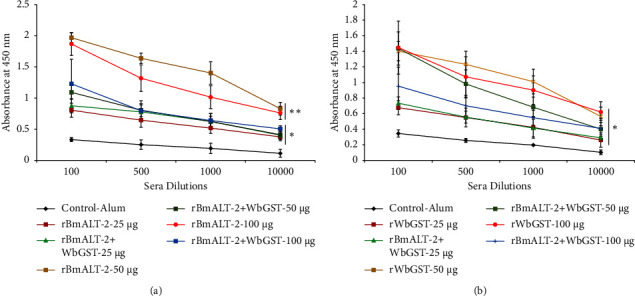
Titer of antigen-specific IgG antibodies in the sera of immunized *Mastomys*. An indirect ELISA was used to determine antibody titer 10 days after the last booster dose. *Mastomys* immunized (i.m.) with different doses (25, 50, and 100 *μ*g) of *Bm*ALT-2, *Wb*GST, *Bm*ALT-2 + *Wb*GST, and alum. Titers of (a) anti-r*Bm*ALT-2 and (b) anti-r*Wb*GST IgG antibodies. Each data point represents mean ± SD, *n* = 5–7 per group. ^*∗*^*p* < 0.05, ^*∗∗*^*p* < 0.005 compared to control-alum group as analyzed by Kruskal–Wallis test, followed by the Bonferroni post hoc test multiple comparison test.

All immunized groups showed high antigen-specific IgG antibody titers after the last dose of the vaccination cycle (Figure 2). The *Mastomys* immunized with 50 or 100 *μ*g r*Bm*ALT-2 generated a significant (*p* < 0.005) IgG antibody titer compared with the *Mastomys* immunized with 25 *μ*g r*Bm*ALT-2 alone and all formulations of r*Bm*ALT-2 + r*Wb*GST (Figure 2(a)). *Mastomys* immunized with 25, 50, or 100 *μ*g r*Wb*GST alone and in combination with r*Bm*ALT-2 generated a substantially higher (*p* < 0.005) IgG antibody titer than control *Mastomys* (Figure 2(b)).

### 3.2. All Immunized *Mastomys* Showed Different Antigen-Specific IgG Isotype Levels

**Figure 3:**
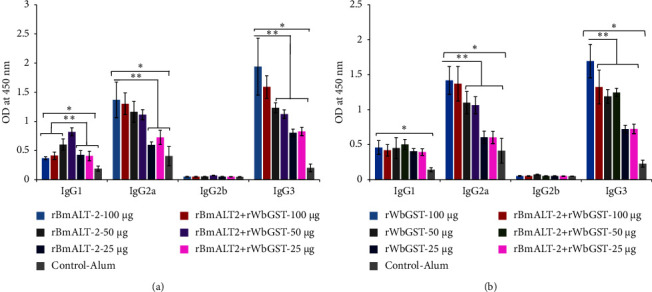
Levels of antigen-specific IgG isotypes in the sera of immunized *Mastomys*. An indirect ELISA was used to determine antigen-specific IgG isotypes (IgG1, IgG2a, IgG2b, and IgG3) 10 days after the last booster dose. *Mastomys* immunized (i.m.) with different doses (25, 50, and 100 *μ*g) of r*Bm*ALT-2, r*Wb*GST, r*Bm*ALT-2 + r*Wb*GST, and alum. (a) Anti-r*Bm*ALT-2 and (b) anti-r*Wb*GST IgG isotypes. Each data point represents mean ± SD, *n* = 5–7 per group. ^*∗*^*p* < 0.05, ^*∗∗*^*p* < 0.005 compared to control-alum group as analyzed by Kruskal–Wallis test, followed by the Bonferroni post-test multiple comparison test.

The serum (1 : 100 diluted) isotype profile showed that all immunized *Mastomys* had significantly (*p* < 0.005) higher IgG1, IgG2a, and IgG3 isotype levels than the control *Mastomys* (Figure 3). There was a progressive increase in IgG2a and IgG3 levels with the increasing dose of test antigens (Figure 3). The *Mastomys* immunized with 50 *μ*g r*Bm*ALT-2 alone and in combination with r*Wb*GST showed significantly higher IgG1 levels (*p* < 0.005) compared with *Mastomys* immunized with 25 or 100 *μ*g r*Bm*ALT-2 alone or the combined r*Bm*ALT-2 + r*Wb*GST immunizations (Figure 3(a)). In contrast, immunization of Mastomys with a 100 *μ*g dose of r*Bm*ALT-2, r*Wb*GST or the combination r*Bm*ALT-2 + r*Wb*GST with alum showed significantly higher IgG2a and IgG3 levels (*p* < 0.005) compared with the 25 or 50 *μ*g antigen-immunized group (Figure 3).

### 3.3. ADCC Assay Showed Larvicidal Activity by Antigen-Specific Antibodies in the Sera of Immunized *Mastomys*

The serum of immunized *Mastomys*, with different doses, 25, 50, or 100 *μ*g r*Bm*ALT-2, r*Wb*GST or their combination, promoted PEC adherence to the L3. All sera of the immunized groups showed significantly higher ADCC than that of the control group; 50 *μ*g r*Bm*ALT-2 induced the highest protection (Table 1). Furthermore, regardless of the antigen dose, only *Mastomys* sera immunized with r*Bm*ALT-2 had the highest activity against L3 (*p* < 0.005) compared to other test antigens.

### 3.4. Diffusion Chamber Assay Showed Significant Prophylactic Efficacy of Antigens

**Figure 4:**
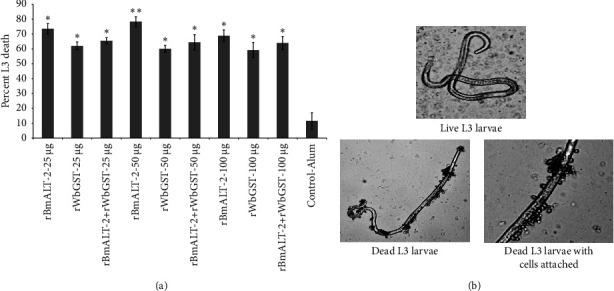
Percent protection in immunized *Mastomys* was calculated by determining the percent larval death. (a) *Mastomys* were immunized i.m. at 4 weeks of intervals with different doses (25, 50, and 100 *μ*g) of r*Bm*ALT-2, r*Wb*GST, r*Bm*ALT-2 + r*Wb*GST, and alum. Ten days after the last immunization, 20 live L3 of *B. malayi* placed in a micropore chamber were surgically implanted into the peritoneal cavity of each *Mastomys*. Chambers were removed aseptically after 48 h, and the L3 death was determined. (b) Several cells were found attached to the dead larvae in the vaccinated *Mastomys*. However, no cells were attached to the live larvae collected from *Mastomys* immunized with alum only. Each bar represents the mean ± SD, *n* = 5–7 per group. ^*∗*^*p* < 0.05, ^*∗∗*^*p* < 0.005 compared to control-alum group as analyzed by Kruskal–Wallis test, followed by the Bonferroni post hoc test multiple comparison test.

All immunized groups induced significantly higher (*p* < 0.005) larval death compared with the control group (Figure 4(a)). When compared with the control group, *Mastomys* immunized with 25 *μ*g r*Bm*ALT-2, r*Wb*GST, and their combination induced 75%, 64%, and 67% protection (*p* < 0.005), respectively. While 50 *μ*g r*Bm*ALT-2 provided higher protection with 79%; *p* < 0.005 compared with 50 *μ*g r*Wb*GST or their combination (Figure 4(a)). Cells were found to be attached to the surface of many recovered dead L3 (Figure 4(b)).

### 3.5. Immunized *Mastomys* Exhibited Increased Cellular Immune Response and Antigen-Recall Response

**Figure 5:**
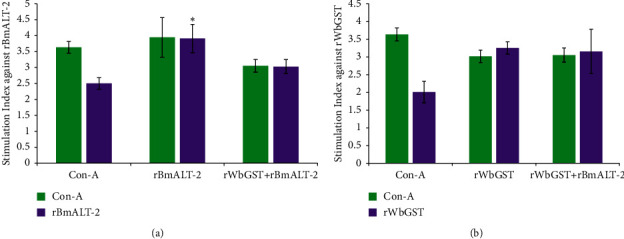
Splenocytes proliferation assay: splenocytes from *Mastomys* administered (i.m.) with (a) r*Bm*ALT-2, r*Bm*ALT-2 + r*Wb*GST, (b) r*Wb*GST, r*Bm*ALT-2 + r*Wb*GST, and control-alum were cultured *in vitro* and re-stimulated with either ConA or with respective recombinant proteins, and the effect on the splenocytes proliferation was checked by an MTS assay after 48 h. The data presented is a mean stimulation index (S.I.) ± SD, *n* = 5–7 per group. ^*∗*^*p* < 0.05 compared to control-alum group as analyzed by Kruskal–Wallis test.

The splenocytes collected from r*Bm*ALT-2-immunized animals showed a significantly (*p* < 0.05) higher proliferative response compared with those from the control group with a stimulation index of SI) 3.91 ± 0.44 vs. 2.50 ± 0.17 when measured by the MTS assay (Figure 5(a)). Splenocytes collected from r*Wb*GST immunized animals showed higher SI compared with those from the control group 3.26 ± 0.17 vs. 2.01 ± 0.30 when measured by MTS assay (Figure 5(b)). The splenocytes from *Mastomys* immunized with r*Bm*ALT-2 + r*Wb*GST responded to both r*Bm*ALT-2 SI 3.02 ± 0.22 and r*Wb*GST SI 3.15 ± 0.62, indicating a strong cellular immune response against these antigens in *Mastomys* vaccinated with r*Bm*ALT-2 + r*Wb*GST (Figure 5).

**Figure 6:**
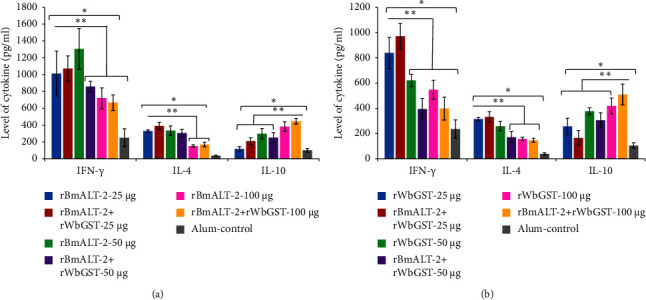
Levels of secreted cytokines (IFN-*γ*, IL-4, and IL-10) in the culture supernatants of spleen cells stimulated for 72 h at 37°C in 5% CO_2_ with (a) r*Bm*ALT-2 or (b) r*Wb*GST were measured using an ELISA. The concentration of each cytokine represented as pg/mL; bar represents the mean ± SD, *n* = 5–7 per group. ^*∗*^*p* < 0.05, ^*∗∗*^*p* < 0.005 compared to control-alum group as analyzed by Kruskal–Wallis test, followed by the Bonferroni post hoc test multiple comparison test.

The restimulated splenocytes of *Mastomys* immunized with 25 or 50 *μ*g r*Bm*ALT-2 alone or in combination with r*Wb*GST significantly released increased (*p* < 0.005) levels of IFN-*γ* and IL-4 compared with *Mastomys* treated with 100 *μ*g r*Bm*ALT-2 or in combination with r*Wb*GST. *Mastomys* treated with 100 *μ*g r*Bm*ALT-2 alone or in combination with r*Wb*GST released significantly increased IL-10 levels (*p* < 0.005) compared with other r*Bm*ALT-2 only treated groups (Figure 6(a)). *Mastomys* treated with 25 *μ*g r*Wb*GST alone or in combination with r*Bm*ALT-2 showed significantly elevated (*p* < 0.005) levels of IFN-*γ* and IL-4 compared with other rWbGST-only treated groups (Figure 6(b)). In contrast, *Mastomys* treated with 50 or 100 *μ*g of r*Wb*GST alone or in combination with r*Bm*ALT-2 displays increased (*p* < 0.005) IL-10 levels compared with the control group (Figure 6(b)).

## 4. Discussion

The present work focuses on two well-documented and potent vaccine candidates: r*Bm*ALT-2 and r*Wb*GST. Previous studies showed that immunization with these proteins individually can induce significant (*p* < 0.05) protection against filariasis in Jirds and *Mastomys* [6, 7, 18]. Antigen immunogenicity is influenced not only by its physical and biological activities but also by its dose [23]. Several findings have suggested that a modest amount of immunogen may produce cell-mediated immunity (CMI), whereas high doses may elicit humoral immune responses [24–26]. However, some research groups indicate that the type and dose of immunogen utilized mostly regulates the immune response [27, 28]. Hence, this study used purified r*Bm*ALT-2 and r*Wb*GST at different doses, 25, 50, and 100 *μ*g, since administering large doses may induce a systemic immune response [29].

Using an *in vitro* and *in vivo*, ADCC and diffusion chamber, approach for this investigation, we found higher protective efficacy with 50 *μ*g r*Bm*ALT-2 than with 25 and 100 *μ*g r*Bm*ALT-2, all doses of r*Wb*GST or the combinations of r*Bm*ALT-2 + r*Wb*GST when alum was used as an adjuvant against *B. malayi* L3. Combination of these two antigens induced approximately 64–68% protection against *B. malayi* L3 *in vitro* [2, 10, 30, 31]. In our study, r*Bm*ALT-2 induced the highest protective efficacy (69–80% *in vitro*) regardless of the dose administered.

ADCC is thought to be an essential immunological mechanism in animals and is primarily responsible for circulating parasite disappearance [32–34]. *In vitro*, cytotoxicity against microfilaria and infective larvae is caused by antibody and complement-mediated effector ADCC pathways [35]. In our *in vitro* study, antisera produced against these two antigens (*Bm*ALT-2 or *Wb*GST) elicited cytotoxicity against L3 within 48 hr. 50 *μ*g of anti-r*Bm*ALT-2 (82% protection) serum increased PEC's adherence to L3 as compared to anti-r*Wb*GST (65% protection) or anti-r*Bm*ALT-2 + r*Wb*GST (69% protection) serum. Similarly, 25 or 100 *μ*g anti-r*Bm*ALT-2 (69–75% protection) serum showed higher cell adhesion than anti-r*Wb*GST (64–65% cytotoxicity) or anti-r*Bm*ALT-2 + r*Wb*GST (67-68% protection) serum.

The *in vivo* diffusion chamber approach, which is performed in a limited physiological environment, has been utilized to investigate parasitic survival, development, and the host effector mechanism. Some experiments using *B. malayi* have been used to show comparable results between *in vitro* and *in vivo assays* [11, 36]. Macrophage participation in cytotoxicity has been verified against different filarial parasites, such as *Dipitelonema setariosum* adults [37] and the L3 of *Litomosoides sigmodontis* [38]. In the current study, we discovered that the *in vivo* diffusion chamber cytotoxicity results were consistent with those obtained from the *in vitro* ADCC test. The i*n vivo*, diffusion chamber experiment showed 78%, 59%, and 65% protective efficacy with 50 *μ*g r*Bm*ALT-2, r*Wb*GST, and their combination, respectively. Similarly, *Mastomys* immunized with 25 or 100 *μ*g r*Bm*ALT-2 showed higher larval killing (approximately 68–73%) than r*Wb*GST or the combination r*Bm*ALT-2 + r*Wb*GST (61–65%). *Mastomys* immunized intramuscularly with 50 *μ*g r*Bm*ALT-2 showed better protective efficacy than other immunogens, both *in vitro* and *in vivo*. Recombinant *Bm*ALT-2 is one of the filarial antigens that can provide substantial protection against L3 [8, 36, 39, 40]. Although the exact mechanism of r*Bm*ALT-2 protein is unknown, studies indicate that it may be crucial for host immunomodulation [38, 41].

Notably, the r*Bm*ALT-2 + r*Wb*GST combination showed higher and lower protective efficacy (65%) than r*Wb*GST alone (61%) and r*Bm*ALT-2 alone (73%) in *vivo*. This study does not rule out the potential that an instantaneous association among these two antigens (r*Bm*ALT-2 and r*Wb*GST) may reduce r*Bm*ALT-2 immunogenicity. r*Wb*GST may have concealed the antigenic epitopes on r*Bm*ALT-2, reducing r*Bm*ALT-2 efficacy in conjugation vaccination [30]. A study on malaria examined the protective potential of two recombinant *Plasmodium yoelli* merozoite surface proteins (MSPs), namely, PyMSP-8 and PyMSP-142, which has shown a similar result. When mice were treated with a combination of these antigens, the immunogenicity of PyMSP-142 decreased [42]. In a filarial study in Jirds, immunization by conjugating r*Bm*TGA and r*Bm*ALT-2 showed merely 47% protection against *B. malayi* L3 [30]. Therefore, selecting antigens for a successful combination is essential, as not every combination yields a positive outcome.

The T-cell response, typically associated with elevated antibody levels, was investigated using IgG isotypes and cytokine profiles. The result indicated that *Mastomys* immunized with 50 or 100 *μ*g r*Wb*GST or r*Bm*ALT-2 or combination of both antigens (r*Bm*ALT-2 + r*Wb*GST) had significant levels of IgG1, IgG2a, and IgG3 antibodies, indicating Th1 and Th2-type reaction. IgG1 and IgG2a isotypes in mice are involved in complement fixation and binding to protein antigens, while IgG3 is involved in carbohydrate-containing epitope identification [36, 41]. Mouse immune complexes with IgG2a and IgG3 can attach to Fc*γ*RI and initiate receptor-mediated responses [36, 43]. These isotypes also contribute to the immune response of vaccines and the *in vitro* reactions (ADCC) against invading pathogens.

Antigen-specific cytokine profiles of *Mastomys* vaccinated with either *Bm*ALT-2 or *Wb*GST or conjugation of both antigens had increased IL-4, IL-10, and IFN-*γ* levels, indicating a Th1 and Th2 immune response. Moreover, 50 *μ*g *Bm*ALT-2 only and in combination with r*Wb*GST induced high IL-10 secretion. IgG1 and IgG3 antibody responses are stimulated explicitly by Th2 cell-produced IL-4 and IL-10, whereas increased IFN-*γ* reflects the Th1 immune response [44, 45]. Our findings indicate that vaccine candidates and their combination elicited mixed Th1/Th2 immune responses. Some research findings suggest that Th2 immune responses contribute to intestinal helminth resistance [45], although applying these findings to tissue-dwelling nematodes, like LF [45], is challenging. The Th1-type immune response is essential to stimulate immune protection against filarial infection [45]. Intramuscular immunization with 50 *μ*g r*Bm*ALT-2 with alum against filarial infection is associated with elevated IFN-*γ*, IL-4, and IL-10 levels and protective Th1 and Th2 immune responses.

In conclusion, *Mastomys* immunized four times at a 4-week interval with 50 *μ*g r*Bm*ALT-2 plus alum as the adjuvant showed an enhanced total IgG antibody titer levels. The isotype profile showed increases in IgG2a, followed by IgG3 and IgG1 levels, demonstrating a mixed Th1 and Th2 response. Immunization with r*Bm*ALT-2 plus alum also significantly increased levels of IFN-*γ*, IL-4, and IL-10 in the spleen cells stimulated *ex vivo*., indicating a mixed Th1 and Th2 response. Moreover, the immunization presented higher protection of approximately 80% against L3 larvae in *in vitro* ADCC and *in vivo* diffusion chamber assay. Therefore, 50 *μ*g/intramuscular dose of r*Bm*ALT-2 plus alum as the adjuvant can be used as an effective immunization dose for human LF to explore further preclinical and mechanistic studies.

## Figures and Tables

**Table 1:** *In vitro* ADCC assay.

Immunization groups	Percent protection
Control-alum	11.81 ± 1.29
25 *μ*g r*Bm*ALT-2	75.96 ± 1.36^*∗*^
25 *μ*g r*Wb*GST	64.10 ± 3.63^*∗*^
25 *μ*g r*Bm*ALT-2 + r*Wb*GST	67.95 ± 1.81^*∗*^
50 *μ*g r*Bm*ALT-2	80.95 ± 3.36^*∗∗*^
50 *μ*g r*Wb*GST	65.15 ± 2.14^*∗*^
50 *μ*g r*Bm*ALT-2 + r*Wb*GST	68.18 ± 6.42^*∗*^
100 *μ*g r*Bm*ALT-2	69.69 ± 4.28^*∗*^
100 *μ*g r*Wb*GST	63.33 ± 4.71^*∗*^
100 *μ*g r*Bm*ALT-2 + r*Wb*GST	68.33 ± 2.35^*∗*^

*B. malayi* L3 were incubated for 48 h with normal peritoneal exudate (PECs) cells and with the pooled sera (50 *μ*L) of immunized (25, 50, or 100 *μ*g rBmALT-2 or rWbGST or their combination) or control group of the Mastomys. The percentages of protection were calculated from total, live, and dead larvae. Data represent the mean ± SD from different sets of experiments (in duplicates), *n* = 5–7/groups; the Kruskal–Wallis test and the Bonferroni multiple comparisons test were used to examine the data, compared to the control-alum group. The percentages of protection were calculated from total, live, and dead larvae. Data represent the mean ± SD from different sets of experiments (in duplicates), *n* = 5–7/groups; the Kruskal–Wallis test and the Bonferroni multiple comparisons test were used to examine the data. ^*∗*^*p* < 0.05, ^*∗∗*^*p* < 0.005 compared to the control-alum group.

## Data Availability

The data used to support the findings of the study are available from the authors upon request.

## References

[1] Mondiale de la Santé (2019). Global programme to eliminate lymphatic filariasis: progress report, 2018–programme mondial pour l’élimination de la filariose lymphatique: rapport de situation. *Weekly Epidemiological Record*.

[2] Anand S. B., Kodumudi K. N., Reddy M. V., Kaliraj P. (2011). A combination of two Brugia malayi filarial vaccine candidate antigens (BmALT-2 and BmVAH) enhances immune responses and protection in jirds. *Journal of Helminthology*.

[3] Sunish I. P., Munirathinam A., Kalimuthu M., Kumar V. A., Tyagi B. K. (2014). Persistence of lymphatic filarial infection in the paediatric population of rural community, after six rounds of annual mass drug administrations. *Journal of Tropical Pediatrics*.

[4] Bhattacharjee J. (2016). Mass drugs administration in India-a failure story. *Epidemiology: Open Access*.

[5] Dyson L., Stolk W. A., Farrell S. H., Hollingsworth T. D. (2017). Measuring and modelling the effects of systematic non-adherence to mass drug administration. *Epidemics*.

[6] Gupta S., Bhandari Y. P., Reddy M. V., Harinath B. C., Rathaur S. (2005). Setaria cervi: immunoprophylactic potential of glutathione-S-transferase against filarial parasite brugia malayi. *Experimental Parasitology*.

[7] Anandharaman V., Dakshinamoorthy G., Gnanasekar M., Reddy M. V. R., Kalyanasundaram R. (2009). Evaluation of wuchereria bancrofti GST as a vaccine candidate for lymphatic filariasis. *PLoS Neglected Tropical Diseases*.

[8] Gregory W. F., Atmadja A. K., Allen J. E., Maizels R. M. (2000). The abundant larval transcript-1 and-2 genes of brugia malayi encode stage-specific candidate vaccine antigens for filariasis. *Infection and Immunity*.

[9] Gnanasekar M., Rao K. V., He Y. X. (2004). Novel phage display-based subtractive screening to identify vaccine candidates of Brugia malayi. *Infection and Immunity*.

[10] Anand S. B., Murugan V., Prabhu P. R., Anandharaman V., Reddy M. V. R., Kaliraj P. (2008). Comparison of immunogenicity, protective efficacy of single and cocktail DNA vaccine of Brugia malayi abundant larval transcript (ALT-2) and thioredoxin peroxidase (TPX) in mice. *Acta Tropica*.

[11] Dakshinamoorthy G., Samykutty A. K., Munirathinam G., Reddy M. V. R., Ramaswamy K. (2013). Multivalent fusion protein vaccine for lymphatic filariasis. *Vaccine*.

[12] Thirugnanam S., Pandiaraja P., Ramaswamy K. (2007). Brugia malayi: comparison of protective immune responses induced by Bm-alt-2 DNA, recombinant Bm-ALT-2 protein and prime-boost vaccine regimens in a jird model. *Experimental Parasitology*.

[13] Gomez-Escobar N., Gregory W. F., Britton C. (2002). Abundant larval transcript-1 and-2 genes from brugia malayi: diversity of genomic environments but conservation of 5′ promoter sequences functional in caenorhabditis elegans. *Molecular and Biochemical Parasitology*.

[14] Dzik J. (2006). Molecules released by helminth parasites involved in host colonization. *Acta Biochimica Polonica*.

[15] Porthouse K. H., Chirgwin S. R., Coleman S. U., Taylor H. W., Klei T. R. (2006). Inflammatory responses to migrating brugia pahangi third-stage larvae. *Infection and Immunity*.

[16] Andure D., Pote K., Khatri V., Amdare N., Padalkar R., Reddy M. V. R. (2016). Immunization with Wuchereria bancrofti glutathione-S-transferase elicits a mixed Th1/Th2 type of protective immune response against filarial infection in mastomys. *Indian Journal of Clinical Biochemistry*.

[17] Rathaur S., Yadav M., Gupta S., Anandharaman V., Reddy M. V. (2008). Filarial glutathione-S-transferase: a potential vaccine candidate against lymphatic filariasis. *Vaccine*.

[18] Ramachandran S., Kumar M. P., Rami R. M. V. (2004). The larval specific lymphatic filarial ALT‐2: induction of protection using protein or DNA vaccination. *Microbiology and Immunology*.

[19] Pain Assigning USDA (2024). *Guidelines for Preparing USDA Annual Reports and Assigning USDA Pain and Distress Categories*.

[20] National Institute of Health (2015). *United States Government Principles for the Utilization and Care of Vertebrate Animals Used in Testing, Research, and Training Revised 2015*.

[21] Suzuki T., Seregeg I. G. (1979). A mass dissection technique for determining infectivity rate of filariasis vectors. *Japanese Journal of Experimental Medicine*.

[22] Anugraha G., Madhumathi J., Prince P. R. (2015). Chimeric epitope vaccine from multistage antigens for lymphatic filariasis. *Scandinavian Journal of Immunology*.

[23] Parish C. R., Liew F. Y. (1972). Immune response to chemically modified flagellin: III. Enhanced cell-mediated immunity during high and low zone antibody tolerance to flagellin. *The Journal of Experimental Medicine*.

[24] Bretscher P. A., Wei G., Menon J. N., Bielefeldt-Ohmann H. (1992). Establishment of stable, cell-mediated immunity that makes susceptible mice resistant to leishmania major. *Science*.

[25] Power C. A., Wei G., Bretscher P. A. (1998). Mycobacterial dose defines the Th1/Th2 nature of the immune response independently of whether immunization is administered by the intravenous, subcutaneous, or intradermal route. *Infection and Immunity*.

[26] Lövgren-Bengtsson K. (1998). 6 Preparation and use of adjuvants. *Immunology of Infection*.

[27] Pfeiffer C., Stein J., Southwood S., Ketelaar H., Sette A., Bottomly K. (1995). Altered peptide ligands can control CD4 T lymphocyte differentiation in vivo. *The Journal of Experimental Medicine*.

[28] Chaturvedi P., Yu Q., Southwood S., Sette A., Singh B. (1996). Peptide analogs with different affinities for MHC alter the cytokine profile of T helper cells. *International Immunology*.

[29] López‐Monteon A., Ramos‐Ligonio A., Pérez‐Castillo L., Talamás‐Rohana P., Luis Rosales‐Encina J. (2003). Specific antibody immune response against the parasitic portion of a glutathione‐S‐transferase fusion protein. *The FASEB Journal*.

[30] Vanam U., Pandey V., Prabhu P. R., Dakshinamurthy G., Rami Reddy M. V., Kaliraj P. (2009). Evaluation of immunoprophylactic efficacy of brugia malayi transglutaminase (BmTGA) in single and multiple antigen vaccination with BmALT-2 and BmTPX for human lymphatic filariasis. *The American Journal of Tropical Medicine and Hygiene*.

[31] Samykutty A., Dakshinamoorthy G., Kalyanasundaram R. (2010). Multivalent vaccine for lymphatic filariasis. *Procedia in vaccinology*.

[32] Mehta K., Sindhu R. K., Subrahmanyam D., Nelson D. S. (1980). IgE-dependent adherence and cytotoxicity of rat spleen and peritoneal cells to litomosoides carinii microfilariae. *Clinical and Experimental Immunology*.

[33] Chandrashekar R., Rao U. R., Subrahmanyam D. (1985). Serum dependent cell‐mediated immune reactions to brugia pahangi infective larvae. *Parasite Immunology*.

[34] Chandrashekar R., Rao U. R., Parab P. B., Subrahmanyam D. (1985). Brugia malayi: serum dependent cell-mediated reactions to microfilariae. *The Southeast Asian Journal of Tropical Medicine and Public Health*.

[35] Sim K. B., Kwa B. H., Mak J. W. (1982). Immune responses in human brugia malayi infections: serum dependent cell-mediated destruction of infective larvae in vitro. *Transactions of the Royal Society of Tropical Medicine and Hygiene*.

[36] Kalyanasundaram R., Balumuri P. (2011). Multivalent vaccine formulation with BmVAL-1 and BmALT-2 confer significant protection against challenge infections with brugia malayi in mice and jirds. *Research and Reports in Tropical Medicine*.

[37] Nelson D. S., Subrahmanyam D., Rao Y. V., Mehta K. (1976). Cellular morphology in pleural exudate of albino rats infected with Litomosoides carinii. *Transactions of the Royal Society of Tropical Medicine and Hygiene*.

[38] Maizels R. M., Blaxter M. L., Scott A. L. (2001). Immunological genomics of Brugia malayi: filarial genes implicated in immune evasion and protective immunity. *Parasite Immunology*.

[39] Sharmila S., Christiana I., Kiran P., Reddy M. V., Kaliraj P. (2011). The adjuvant-free immunoprotection of recombinant filarial protein abundant larval transcript-2 (ALT-2) in mastomys coucha and the immunoprophylactic importance of its putative signal sequence. *Experimental Parasitology*.

[40] Paul R., Ilamaran M., Khatri V., Amdare N., Reddy M. V., Kaliraj P. (2019). Immunological evaluation of fusion protein of brugia malayi abundant larval protein transcript-2 (BmALT-2) and Tuftsin in experimental mice model. *Parasite Epidemiology and Control*.

[41] Akiyama Y., Lubeck M. D., Steplewski Z., Koprowski H. (1984). Induction of mouse IgG2a-and IgG3-dependent cellular cytotoxicity in human monocytic cells (U937) by immune interferon. *Cancer Research*.

[42] Shi Q., Lynch M. M., Romero M., Burns J. M. (2007). Enhanced protection against malaria by a chimeric merozoite surface protein vaccine. *Infection and Immunity*.

[43] Leu R. W., Robinson C. J., Wiggins J. A., Shannon B. J., Rummage J. A., Horn M. J. (1988). Photometric assays for FcRI-dependent binding, phagocytosis, and antibody-dependent cellular cytotoxicity mediated by monomeric IgG*γ*2a in murine peritoneal macrophages. *Journal of Immunological Methods*.

[44] Lawrence R. A. (2001). Immunity to filarial nematodes. *Veterinary Parasitology*.

[45] Brunet L. R., Finkelman F. D., Cheever A. W., Kopf M. A., Pearce E. J. (1997). IL-4 protects against TNF-alpha-mediated cachexia and death during acute schistosomiasis. *The Journal of Immunology*.

